# A Description of an Improved Screw Tourniquet

**Published:** 1800

**Authors:** W. Simmons

**Affiliations:** Member of the Corporation of Surgeons of London; and Senior Surgeon to the Manchester Infirmary.


### II. *A description of an improved screw Tourniquet. By the Same.*

THE great importance of the tourniquet in chirurgical practice, is too obvious to admit of discussion.

The first form of this instrument, consisted of a loose bandage, for the purpose of surrounding the limb; and of a cylindrical stick, a few inches in length; which, being passed through the bandage, was ordered to be twisted, until such a degree of compression was produced on the main artery, by bolsters placed in its course, as to stop the flux of blood, should it chance to be wounded or divided.

In addition to these materials, a strong square piece of leather was employed, and perforated on two sides, for the transmission of the ends of the roller, to guard the limb from injury by the stick, and to give a firmer purchase; and, as the power was derived from the latter, it has been denominated the stick-tourniquet.

The continual attendance requisite to prevent a tourniquet thus constructed from untwifting, would prefent a ferious objection to it; for which, and fome other reafons, M. Petit, a furgeon of great eminence in Paris, fubftituted a fpiral fcrew inftead of the ftick. Petit's inftrument, however, was liable to objections; and Morand, Heifter, and others, propofed fome alterations; which were again fuperfeded by the improvement of our countryman Freke.

Notwithftanding the improved ftate of Petit's tourniquet, it has been found to flip occafionally; by which the roller would become flack, and the hemorrhage would be renewed.

Befides the inconvenience of flipping (a defect appertaining to the ftructure of the inftrument) if the male fcrew fhould be worn from frequent ufe, or become greafy, from negligence in cleaning, it will require nearly as much attention from the affiftant, as the ftick-tourniquet. The inftrument has been made too flender alfo, for the compreffion of a large mufcular limb; fo that, in fome inftances, it has broken, to the great perplexity of the operator, and extreme hazard of the patient.

The principal objects to be held in view, in conftructing conſtructing the tourniquet, appear to be two; firſt, that it ſhall be made ſo ſtrong, as not to riſk a want of power, under any circumſtances; and, ſecondly, to prevent the ſcrew from yielding, and the bandage from becoming looſe.

The firſt intention will be accompliſhed, I apprehend, by conſtructing Petit's tourniquet, as improved by Freke, which is that in common uſe, of nearly double ſtrength; and the ſecond, by making a tranſverſe male and female ſcrew, to paſs horizontally through the upper plate, ſo that the point of the male ſcrew, ſhall reſt upon the inner thread of the perpendicular ſcrew.

When the tourniquet ſhall have been placed on a limb, in the uſual way, and the perpendicular ſcrew ſhall have been tightened, at the diſcretion of the operator; the tranſverſe ſcrew ſhould be turned till it meets with the reſt juſt mentioned; when it will be found to have made the inſtrument perfectly ſecure. The contrivance is ſo ſimple, as to require no further direction for its management; for it is plain, that the tranſverſe ſcrew muſt be unturned to free it from preſſure, either on the inner or outer thread of the perpendicular

ſcrew; and then, in its principle, it becomes merely the common tourniquet.

The tranſverſe male ſcrew ſhould be made of iron, and, for a ſpace meaſuring the diſtance from the outer to the inner thread of the perpendicular ſcrew, the end ſhould be quite ſmooth, and of a ſize to fill nearly the ſpace intervening between two outer threads of the ſcrew: the other parts ſhould be conſtructed of braſs, as uſual.

The tourniquet thus formed will conſiſt of a tranverſe ſcrew, made of iron, preſſing upon a perpendicular ſcrew, made of braſs; with the further ſecurity, of the ſmooth end of the former, affording a reſt to the under ſurface of the outer thread of the latter. And, as the direction of the force will always be downward, if the inſtrument be properly conſtructed, and of good materials, the greater the preſſure, the more firm and ſecure the inſtrument will be. Another advantage attending it is, that the check-part can ſcarcely be put out of order; and, if at any time faulty, it may be readily mended or replaced.

The annexed figure will better explain the alteration; of theſe Figure VI. (Plate I.) repreſents the whole inſtrument combined; and and Figure VII. the tranſverſe ſcrew ſeparate.

The tourniquet is an inſtrument ſo well known, as to need no further deſcription.

N. B. Mr. Richardſon, cutler, in Mancheſter, makes tourniquets according to theſe directions.

